# New trends in anaphylaxis

**DOI:** 10.1007/s40629-017-0042-y

**Published:** 2017-11-15

**Authors:** Margitta Worm, Gunter Sturm, Jörg Kleine-Tebbe, Ewa Cichocka-Jarosz, Victoria Cardona, Ioana Maris, Sabine Dölle

**Affiliations:** 1 Department of Dermatology and Allergology, Charité - Universitätsmedizin Berlin, Berlin, Germany; 2 University Department of Dermatology and Venereology, Medical University Graz, Graz, Austria; 3 Hanf, Ackermann and Kleine-Tebbe Allergy Practice, Allergy and Asthma Center Westend, Berlin, Germany; 4 Department of Pediatrics, Pulmonology, Allergology, and Dermatology Division, Institute of Pediatrics, Jagiellonian University Medical College, Krakau, Poland; 5 Allergy Section, Department of Internal Medicine, University Hospital Vall d’Hebron, Barcelona, Spain; 6 Department of Paediatrics and ChildHealth, University College Cork, Cork, Ireland

**Keywords:** Anaphylaxis, Hymenoptera venom allergy, Education food allergy, Molecular diagnostics, Emergency care

## Abstract

This review presents the current trends in anaphylaxis management discussed at the fourth International Network for Online-Registration of Anaphylaxis (NORA) conference held in Berlin in April 2017. Current data from the anaphylaxis registry show that Hymenoptera venom, foods, and pharmaceutical drugs are still among the most frequent triggers of anaphylaxis. Rare triggers include chicory, cardamom, asparagus, and goji berries. A meta-analysis on recent trends in insect venom anaphylaxis demonstrated for the first time that, although data on the efficacy of insect venom immunotherapy is limited, the occurrence of severe reactions upon repeated sting events can be prevented and patients’ quality of life improved. Molecular diagnostics of insect venom anaphylaxis have significantly improved diagnostic sensitivity and specificity. Self-treatment of anaphylaxis is of great importance. Recent data from the anaphylaxis registry show an increase (from 23% in 2012 to 29% in 2016) in the use of adrenaline as recommended in the guidelines. A survey on the implementation of guidelines conducted among the centers reporting to the anaphylaxis registry highlights the extent to which the guideline has been perceived and implemented. Reports on a variety of cases in the anaphylaxis registry illustrate the diversity of this potentially life-threatening reaction. Component-resolved diagnostics can help to specify sensitization profiles in anaphylaxis, particularly in terms of the risk for severe reactions. Recent studies on anaphylaxis awareness show that training methods are effective; nevertheless, target groups and learning methods need to undergo further scientific investigation in coming years.

## Introduction

The fourth International conference of the Network for Online-Registration of Anaphylaxis (NORA), held in Berlin on 7th and 8th April 2017, was attended by NORA participants from seven countries and recent data as well as variety of topics relating to anaphylaxis were discussed.

## Current data

**Fig. 1:**
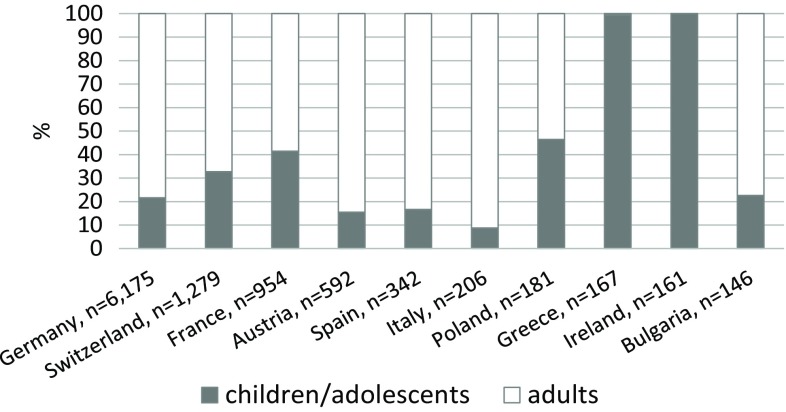
Total numbers and percentages of reports on children and adults in countries participating in the anaphylaxis registry (as of March 2017), *n* = 10,212

Since its inception over 10 years ago, the Anaphylaxis Network has contributed an abundance of data on the triggers and management of anaphylaxis not only in German-speaking countries, but across Europe [1–3]. A total of 10,212 cases from 12 countries were registered between 2007 and 2017. The vast majority of registered cases came from German-speaking countries, followed by data from the French network, as well as Spain, Italy, Poland, Greece, Ireland, and Bulgaria. Individual cases were reported from Brazil (*n* = 8) and Slovenia (*n* = 1). The trend in reported cases shows that between 800 and 1200 cases have been reported annually to the anaphylaxis registry across Europe since 2012. Most countries reported cases in both children and adults. Greece and Ireland were exceptions here in that they reported cases in children only (Fig. 1). The trigger profiles for the reactions reported by the individual countries are specific and age-dependent. For example, food-related anaphylaxis is seen primarily in Ireland and Greece, followed by Spain and France, whereas Hymenoptera venom is a main trigger of severe allergic reactions in Germany, Austria, and Italy.

The anaphylaxis registry is also able to provide data on rare triggers of anaphylaxis. For example, isolated cases triggered by chicory (Munich, Germany), cardamon (Homburg, Germany), asparagus (Nancy, France), or goji berries (Munich, Germany) were recently registered (Table 1).

**Table 1:** Case reports on rare elicitors of food-induced anaphylaxis

	Chicory	Cardamon	Asparagus	Goji berries
Age (in years)	47	63	44	51
Sex	Female	Male	Male	Male
Severity according to Ring and Messmer	II	III	I	III
Center	LMU, Munich, Germany	University Hospital Saarland, Homburg/Saar, Germany	Allergy Vigilance, Nancy, France	LMU, Munich, Germany
Country	Germany	Germany	France	Germany
Year of reaction	2013	2010	2011	2010
Interval between allergen contact and reaction (in min)	0–10	>120	>120	0–10
Type of reaction	First reaction	First reaction	Recurrent reaction	First reaction

## Hymenoptera venom anaphylaxis: current trends

### Immunotherapy and risk factors

Recent data analyses show that Hymenoptera venom immunotherapy to treat Hymenoptera venom-allergic patients is highly effective and has a fast onset of action [4]. A meta-analysis recently published on behalf of the European Academy of Allergy and Clinical Immunology (EAACI) yielded similar results [5]. This latter analysis concluded that, although data on the efficacy of insect venom immunotherapy are limited, they nevertheless suggest that severe reactions upon following or future sting events can be prevented and the quality of life of patients positively affected by immunotherapy [5].

Two other important aspects of Hymenoptera venom allergy include risk factors for treatment and side effects and risk factors for the recurrence of reactions following therapy. Earlier studies showed that Hymenoptera immunotherapy confers a 3.1- to 6‑fold higher risk for therapy-related side effects [6–8]. A further study showed that high specific IgE levels or high skin test reactivity do not make it possible to predict the risk for side effects [6, 9]. The evidence relating to the use of beta-blockers and angiotensin-converting enzyme (ACE) inhibitors in the setting of Hymenoptera venom immunotherapy remains controversial [10, 11]. Although, in principle, beta-blockers do not appear to increase the rate of side effects, they may have an effect on the severity reactions [12]. With regard to ACE inhibitors, a handful of studies show that these agents represent a risk factor for severe reactions during therapy [10, 13]. With regard to the duration of immunotherapy, the published data suggest that 5‑year therapy is preferable and that side effects under treatment, as well as extremely strong reactions prior to treatment initiation, are an indication that a reaction is highly likely following treatment.

### Molecular diagnostics of Hymenoptera venom anaphylaxis

Molecular diagnostics of Hymenoptera venom allergy have evolved over the last few years, and cross-reactive allergens in Hymenoptera venom allergy were recently identified [14]. It has been possible to establish the marker allergens Api m 1, Api m 3, Api m 4, and Api m 10 for the bee and Ves v 1 and Ves v 5 for the wasp. Other allergens, such as Api m 2, Ves v 2, Api m 5, Ves v 3, as well as Api m 12 and Ves v 6, have been classified according to current data as indicators of cross-reactivity [14]. Thus, by determining these individual parameters, it is possible to differentiate primary sensitization to bee or wasp venom. The user’s guide to molecular allergy diagnostics [15] recently published by the EAACI provides up-to-date information on this. The fact that the spectrum has broadened to include the compilation of sensitization profiles additionally offers approaches to explain why patients potentially would not respond to Hymenoptera venom immunotherapy. For example, recent data indicate that there are honeybee venom extracts available for immunotherapy that contain insufficient amounts of Api m 10 and, as such, do not represent an appropriate treatment option for patients predominantly sensitized to Api m 10 [16]. Thus, the use of component-resolved diagnostics in Hymenoptera venom-allergic individuals can help to improve patients’ response to treatment.

### Self-treatment in Hymenoptera venom allergy

Self-treatment plays an important role in Hymenoptera venom allergy [17]. Other questions include the following: which patients require self-medication and how does one carry out effective training of those affected? The principles of emergency treatment of Hymenoptera venom allergy include the use of drugs for self-medication once the stinger, if present, has been removed. Patients that have already experienced a severe reaction should be issued with an adrenaline autoinjector. This applies in particular to patients with mast cell disease, as well as to those with risk factors for treatment failure—indeed before, during, and after SIT (specific immunotherapy) with Hymenoptera venom. The treatment of choice, intramuscular adrenaline, is based on pharmacokinetic data that show a rapid surge of adrenaline with this mode of administration [18]. A variety of autoinjectors are available in Europe and North America. However, numerous studies have shown that, in reality, adrenaline autoinjectors are only rarely used [19]; moreover, even in emergency departments, they are not always prescribed following an anaphylactic reaction [20]. Experience gained from various training concepts suggest that it is possible to improve the use of adrenaline in the emergency setting.

## EAACI task force on “Implementing the guidelines”

**Fig. 2:**
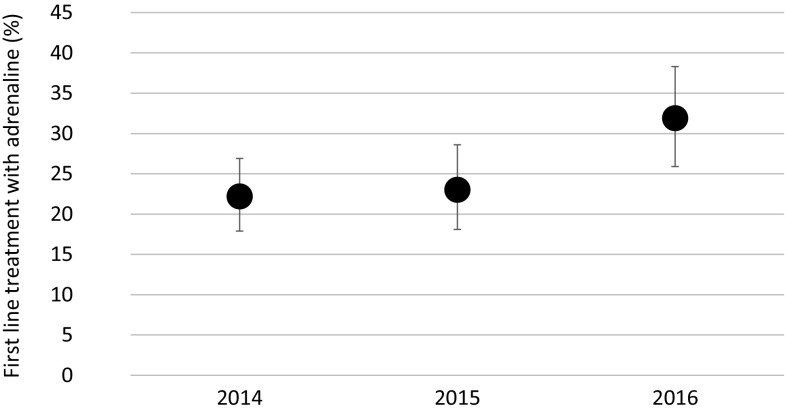
Percentages of first line treatment with adrenaline for severe anaphylactic reactions (severity grade III/IV according to Ring and Messmer, data from all countries from 2014 to 2016)

**Fig. 3:**
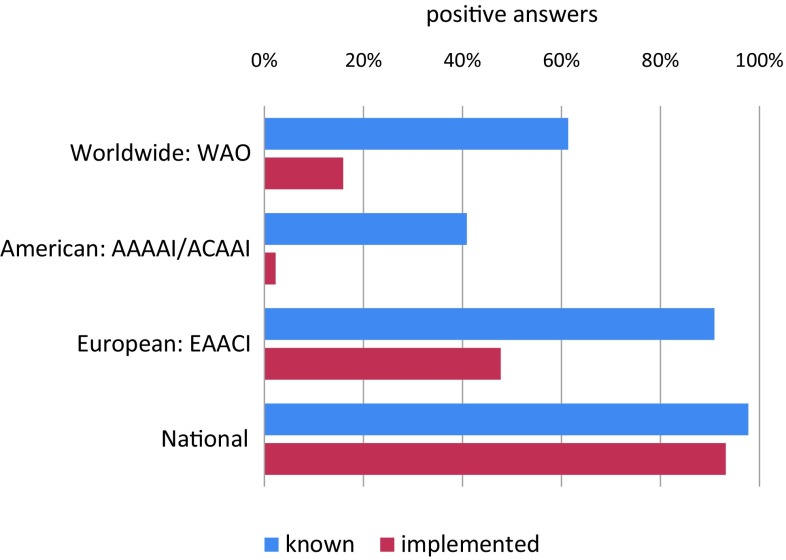
Results of an online survey among NORA members (*n* = 44) of the anaphylaxis registry on anaphylaxis treatment. Question: Which guideline(s) are you familiar with and which one(s) is (are) implemented in your center? *WAO* World Allergy organization, *AAAAI/ACAAI* American Acadamy/College of Allergy, Asthma, and Immunology, *EAACI* European Acadamy of Allergy and Clinical Immunology

Guideline implementation is an important issue and one of the major activities of the EAACI task force. Current data from the anaphylaxis registry point out that adrenaline is still not frequently used even in severe anaphylaxis. However, for the years 2014–2016 (Fig. 2) an increase in the use of intramuscular adrenaline in anaphylaxis patients in line with the current guidelines [21, 22] is reported. A survey of NORA network members on anaphylaxis management at European and German centers revealed good knowledge among personnel regarding the route of administration and dosage of adrenaline, as well as familiarity with the guidelines, at the respective centers (Fig. 3).

The severity score newly developed by the team under M. Worm has been presented as a future project and will be used in an explorative approach by the participating centers in the next phase of the project.

## Case reports from participating centers

### Anti-IgE in Hymenoptera venom immunotherapy

One case report (Dorothea Wieczorek, Hannover, Germany) deals with protective role of anti-IgE therapy in the context of SIT [23]. In this particular case, anti-IgE treatment was successfully performed in the context of Hymenoptera venom immunotherapy in a patient that had experienced a severe reaction in the past. This case report addressed the question of when is the optimal point in time for anti-IgE therapy and whether a single dose can in fact be sufficient to prevent systemic side effects during SIT.

### Anaphylaxis in children

One case described a 3-year-old boy with a severe biphasic anaphylaxis and a selective allergy to Brazil nuts. Despite a serologically positive cross-reaction to hazelnut and walnut (Simoneta Hernández Reyes, Madrid, Spain). A further pediatric case, this time from Germany (Susanne Hämmerling, Heidelberg), involved a 6-year-old boy who experienced severe anaphylaxis following a liver transplant. In addition to the relevance of a possible transfer of anaphylaxis via organ transplantation, tacrolimus administration was also discussed as a potential cause given that, according to the literature, it may interfere with the incidence of food allergy [24, 25].

### Drug-induced anaphylaxis

A case of chlorhexidine-induced anaphylaxis is interesting in view of the announcement issued by the US Food and Drug Administration (FDA) in February 2017 that chlorhexidine can cause severe allergic reactions (Roland Lang, Salzburg, Austria). The active substances in drugs are capable to induce allergies, but additives may also mediate non-Ige dependent hypersensitivities. The onset in an 11-year-old boy with a severe allergic reaction to carmosine (a food coloring) contained in a medication was reported by Tihomir Mustakov (Sofia, Bulgaria). The relevance of additives in general and colorings in food and drugs in particular remains in terms of their capacity to elicit severe allergic reactions still controversial and represents a diagnostic challenge [26].

### Recurrent reactions in mustard and sesame allergy

A 65-year-old man with multiple anaphylactic episodes within 18 months presented for a diagnostic work-up (Macarena Knop, Munich, Germany). A detailed patient history revealed mustard to be a possible cause, given that it had regularly been consumed in the context of the episodes. Positive skin prick testing and positive oral provocation testing confirmed mustard as the trigger of the reactions.

“Open sesame” was a further case of food allergy as the cause of anaphylaxis. This case involved a 62-year-old man who reported recurrent anaphylactic reactions following the consumption of fitness bread rolls, hamburgers, and roasted duck (Sabine Dölle, Berlin, Germany). The diagnostic allergy work-up revealed sesame to be the trigger and, in all likelihood, the oleosins therein to be the allergenic source. Since oleosins do not dissolve readily in water, it is common for both skin testing with native sesame and provocation testing to be negative.

### Abdominal pain and specific immunotherapy

A 37-year-old woman presented due to a sudden-onset abdominal pain during SIT (specific immunotherapy) that persisted despite adrenaline administration (Olaya Álvarez García, Madrid, Spain). An ad hoc ultrasound examination revealed a hemorrhagic ovarian cyst to be the cause of symptoms. This case shows that gynecological symptoms can occur in the setting of Hymenoptera venom immunotherapy, as well as other immunotherapies. The causal mechanism for this is as yet unknown.

### Evidence of a KIT D816V mutation in idiopathic anaphylaxis

A case of an unknown mastocytosis in a woman with idiopathic anaphylaxis and normal tryptase levels, determined by measuring KIT D816V mutations in peripheral blood, was presented. It illustrates the potential of this genetic analysis to identify an occult mastocytosis (Wojciech Francuzik, Berlin, Germany).

## Component-resolved diagnosis in anaphylaxis

Component-resolved allergy diagnosis in anaphylaxis represents a challenge in terms of its complexity and scope. Particular sensitization profiles in food allergy, e. g., lipid transfer protein (LTP) and storage protein sensitizations [27, 34], exhibit a high risk for severe reactions.

Whilst alpha-Gal sensitizations are established as a marker for delayed immediate-type reactions to mammalian meat [28], they can also point to a risk for reactions to certain medications [29]. It is possible that the use of microchips could be particularly helpful in patients with idiopathic anaphylaxis. A study on 110 patients showed that multiple sensitizations to various allergens were present in patients with idiopathic anaphylaxis [30, 34].

## Anaphylaxis awareness

The main questions relating to anaphylaxis awareness include the following: who needs anaphylaxis training, which teaching methods can be used, and what are the specific challenges faced? Target groups for training courses include patients and relatives, school staff, the general public, as well as individuals working in the health system. A number of articles have been published on these topics [31–33], although the success of training in some of the studies can be deemed as only limited.

## Summary and outlook

Especially molecular allergy diagnostics and the treatment of anaphylaxis are on track to improve the management of patients with severe allergic reactions. National and international guidelines and the development of training programs are important features to facilitate their implementation.

The data and collaboration of centers participating in the anaphylaxis registry make an important contribution to this. In the future, the development of an instrument to measure severity should allow a better differentiation of affected patients.

## Abbreviations

EAACI: European Academy of Allergy and Clinical Immunology
FDA: US Food and Drug Administration
IgE: Immunoglobulin E
LTP: Lipid transfer proteins
NORA: Network for Online-Registration of Anaphylaxis
SIT: Specific immunotherapy
